# High-Efficiency Degradation of Orange II by Co_78_Si_8_B_14_/g-C_3_N_4_ Composite Catalyst in a Visible-Light-Assisted Peroxymonosulfate Activation System

**DOI:** 10.3390/ma18081733

**Published:** 2025-04-10

**Authors:** Zhenling Yang, Guofeng Ma, Jun Zhang

**Affiliations:** 1 College of Environment, Shenyang University, Shenyang 110044, China; 2 Shenyang Key Laboratory of Micro-Arc Oxidation Technology and Application, Institute of Innovative Science and Technology, Shenyang University, Shenyang 110044, China; 3 College of Mechanical Engineering, Shenyang University, Shenyang 110044, China

**Keywords:** amorphous alloy, dye degradation, peroxymonosulfate, visible light-assisted, photocatalytic degradation

## Abstract

Peroxynitrite-based advanced oxidation technology has gradually become a research hotspot for degrading dye wastewater due to its high efficiency and environmentally friendly features. Transition metal elements, which are commonly used as catalysts for the activation of persulfates, suffer from problems such as easy deactivation and leaching of metal ions, which limit their practical application. In this study, Co_78_Si_8_B_14_/g-C_3_N_4_ composite catalysts were prepared by wet milling and ball milling methods to investigate their degradation of Orange II dyes by assisting the activation of peroxynitrite under visible light, and the effects of the catalyst ratio, light intensity, and the dosage of catalysts on the degradation performance were investigated. It was shown that the optimum ratio of Co_78_Si_8_B_14_ to g-C_3_N_4_ was 1:3, and the reaction rate constants for the degradation of orange dye by Co_78_Si_8_B_14_/g-C_3_N_4_ + PMS + VIS were 4.3 and 5.37 times higher than those of single g-C_3_N_4_ + PMS and Co_78_Si_8_B_14_ + PMS, respectively. Meanwhile, the composite catalyst also showed good degradation performance for rhodamine B, methyl orange, and methylene blue dyes, and the degradation effect could reach more than 75%. Cyclic stability tests showed that the catalyst maintained a high degradation efficiency of more than 94% over multiple cycles with low ion dissolution concentration. Its high catalytic activity is attributed to the lowest adsorption energy of the composite catalyst to PMS (E_ads_ = −1.97 eV), which facilitates the degradation reaction, while the synergistic effect of g-C_3_N_4_ and Co_78_Si_8_B_14_ promotes the production of ·SO_4_, ·OH, and ·O^2−^. This study provides new ideas for the development of stable and efficient catalysts to expand the synergy between PMS-based and other advanced oxidation technologies.

## 1. Introduction

The efficient degradation of organic pollutants has become a research hotspot in the face of growing environmental pollution problems [1,2]. Orange II, as a typical azo dye, is widely used in industries such as printing and dyeing [3,4]. However, due to its refractory properties, it will cause great harm to the environment if directly discharged without effective treatment [5]. At present, the main degradation methods of Orange II include Fenton oxidation, microbial degradation, photocatalytic degradation, physical adsorption, etc. [6,7,8]. The advanced oxidation technology of peroxymonosulfate (PMS) for the degradation of Orange II has gradually attracted attention in recent years [9]. Its redox potential is similar to that of ozone or hydrogen peroxide and has potential advantages in water treatment, such as being a solid at room temperature, easy to store and transport, and having high stability and water solubility [10,11].

Light-activated PMS technology has unique advantages in the degradation of organic pollutants [12]. This technology can generate active species with strong oxidation ability and can effectively degrade organic pollutants [13]. The team of Professors Liu and Wang proposed a new method for coupling photocatalysis and PMS e-activation [14]. Through light energy, excitation generates photogenerated electrons in the photocatalytic semiconductor to continuously activate PMS, achieving synergistic and efficient performance. The team constructed hydrogel spheres with catalytic functions and proposed the mechanism of activation by light excitation and synergistic PMS. Under light excitation conditions, an internal built-in electric field is generated inside the hydrogel sphere to redistribute the energy barrier and provide a driving force for charge transfer. The integration of photogenerated electrons into the PMS activation system improves the electronic characteristics of the traditional transition metal activation of PMS. It extends the ideas for the development and application of new PMS-activated functional materials in the field of water remediation.

g-C_3_N_4_, as a two-dimensional non-metallic semiconductor polymer, has a wide band gap width, abundant active sites, and low raw material synthesis cost [15,16]. It shows broad application prospects in the field of collaborative photocatalysis and activation of PMS for the degradation of organic pollutants [17]. The compounding of g-C_3_N_4_ can not only overcome the defects of pure carbon nitride, such as low visible light utilization efficiency, small specific surface area, and rapid recombination of photogenerated electron–hole pairs, but also significantly expand its light response range, promote the electron migration of activated PMS, and thereby enhance the catalytic activity [18,19,20].

In this study, the Co_78_Si_8_B_14_/g-C_3_N_4_ nanocomposite catalyst was prepared by the wet milling method. The phase structure and morphology of the composite catalyst were analyzed by characterization methods such as XRD, XPS, SEM-EDS, and TEM. The catalytic performance was investigated by degrading Orange II dye through the light-assisted activation of PMS, and the influence of single factors on the degradation was studied. The stability of the composite catalyst was further judged by cyclic tests and ion leaching rate detection. In addition, EPR and radical capture experiments were used to investigate the active species generated during the catalytic degradation process and to study the degradation mechanism. This study explains the reason for the high degradation performance of the composite catalyst and provides an environmentally friendly and sustainable catalyst for activating PMS to degrade dyes under visible light.

## 2. Experimental Section

### 2.1. Materials Preparation and Characterization

The main reagents used in the experiment are as follows: Orange II (C_16_H_11_N_2_NaO_4_S, AR, Shanghai Yuanye Biotechnology Co., Shanghai, China); sodium persulfate (Na_2_S_2_O_8_, AR, Tianjin Damao Chemical Reagent Factory, Tianjin, China); ethanol absolute (CH_3_CH_2_OH, AR, Tianjin Yongda Chemical Reagent Co., Tianjin, China); 1,4-Benzoquinone (C_6_H_4_O_2_, 97%, Aladdin Biochemical Technology Co., Shanghai, China); Ttert-Butanol (C_4_H_10_O, AR, Tianjin Damao Chemical Reagent Factory); sodium hydroxide (NaOH, AR, Tianjin Comeo Reagent Co., Tianjin, China); hydrochloric acid (HCl, AR, Tianjin Comeo Reagent Co., Tianjin, China); methylene blue (C_16_H_18_N_3_ClS, AR, Shanghai Yuanye Biotechnology Co.); rhodamine B (RhB, AR, CHClN_2_O_3_, Shanghai Yuanye Biotechnology Co.); methyl orange (C_14_H_14_N_3_SO_3_Na, AR, Aladdin Biochemical Technology Co.); and urea (CH_4_N_2_O, AR, Tianjin Damao Chemical Reagent Factory). All the above reagents are analytically pure.

The crystal structure of the samples was characterized using X-ray diffraction (XRD, X’Pert Pro, PANalytical, Almelo, The Netherlands). X-ray photoelectron spectroscopy (XPS, Nexsa G2, Thermo Scientific, Waltham, MA, USA) characterizes elemental types and chemical valence states. The surface morphology of the composite catalysts was observed by scanning electron microscope (SEM, S-4800, Hitachi, Tokyo, Japan) and an accompanying electron spectroscopy analyzer (EDS, HITACHI S-4800, Tokyo, Japan) with an accelerating voltage of 5 kV, and elemental analyses were performed on the surface compositions. The microscopic morphology and lattice structure of the samples were tested using a high-resolution transmission electron microscope (TEM, JEM2100, JEOL, Tokyo, Japan) operated at an accelerating voltage of 200 kV. Hydroxyl radicals and superoxide radicals were measured using electron paramagnetic resonance (EPR, A300, Bruker, Berlin, Germany). The EPR detection used a center field of 3500 G, a sweep width of 150 G, a sweep time of 30 s, a microwave power of 3.99 mW, a modulation amplitude of 1 G, and a conversion time of 40 MS at 9.854854 GHz. The specific surface area (BET) of samples was measured using a static nitrogen adsorbent analyzer (JW-BK122F, Jingwei Gao Bo Science and Technology Co., Ltd., Beijing, China).

### 2.2. Preparation of Co_78_Si_8_B_14_/g-C_3_N_4_ Catalyst

(1) Preparation of g-C_3_N_4_

Urea was added to an alumina crucible and then placed in a tube furnace through Ar gas at a calcination temperature of 550 °C, a temperature rate of 10 °C·min^−1^, and a holding time of 2 h. After cooling to room temperature, it was ground to get a yellow powder that is g-C_3_N_4_. In order to avoid agglomeration, it was then dispersed in isopropanol g-C_3_N_4_ powder and underwent ultrasonic stripping for 3–5 h. Finally, it was centrifuged to obtain the precipitate. After washing and drying, graphite-phase carbon nitride nanoflake material was obtained.

(2) Preparation of Co_78_Si_8_B_14_ powder

Co_78_Si_8_B_14_ amorphous alloy powder was prepared by gas atomization. We used cobalt (Co ≥ 99.99%), silicon (Si ≥ 99.9%), and boron (B ≥ 99.5%) at an atomic ratio of 78:8:14, weighing raw materials accurately to ±0.1%. The raw materials were acid-washed to remove oxides, cleaned by alcohol ultrasonic cleaning, and dried. The raw materials were melted in a vacuum induction melting furnace with argon protection (purity ≥ 99.999%) and a melting temperature of 1500–1600 °C. Melting was repeated 3–4 times to ensure the uniformity of composition. When the melt flows through the nozzle, it is broken into micron-sized droplets by high-pressure argon gas and cooled rapidly in the atomization tower. Amorphous Co_78_Si_8_B_14_ powder is prepared by spraying the alloy melt through the nozzle with a diameter of 0.8 mm by high pressure under the protection of high-purity argon gas.

(3) Preparation of Co_78_Si_8_B_14_/g-C_3_N_4_ composite catalysts

**Figure 1:**
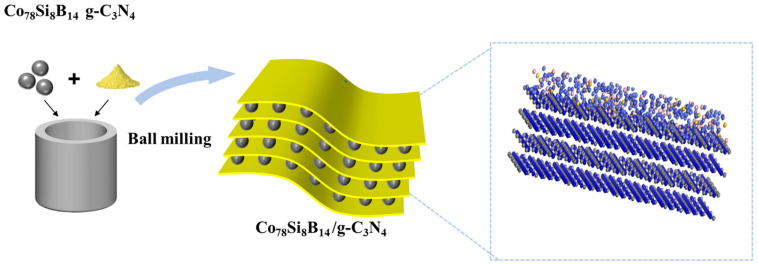
Schematic diagram of Co_78_Si_8_B_14_/g-C_3_N_4_ preparation.

The steps for the preparation of Co_78_Si_8_B_14_/g-C_3_N_4_ composite catalyst by means of wet milling were as follows: Co_78_Si_8_B_14_ amorphous alloy powder with different mass ratios was weighed and mixed with g-C_3_N_4_ powder. The powder mixture was put into a ball milling jar with a ball material ratio of 10:1; anhydrous ethanol was added, and ball milling was carried out for 10 h under an argon protective atmosphere at a rotational speed of 200 r·min^−1^. In order to avoid amorphous alloy crystallization caused by the high temperature during the ball milling process, the ball mill was set to stop for 10 min after every 10 min of operation, and the actual length of the ball milling was 5 h. Finally, the composite catalyst material was dried in a vacuum drying oven at 60 °C for 24 h to obtain the composite catalyst material, as shown in Figure 1.

### 2.3. Degradation Experiment

A certain initial concentration of Orange II solution was prepared in deionized water. A certain amount of amorphous alloy strip material was added to the aqueous solution containing Orange II, and after mechanical stirring for 30 min, Na_2_S_2_O_8_ was added to start the activation reaction when an adsorption–desorption equilibrium was reached between the catalyst and the pollutant molecules. At certain time intervals, 5 mL of the mixed solution was quickly sampled and immediately filtered through a 0.45 μm filter membrane for determination and analysis with a UV-Vis spectrophotometer. Each set of experiments was averaged three times in parallel. The degradation rate was calculated according to Equation (1).

(1) $$\eta =(1-\frac{C}{C_{0}})\times 100\%$$

where *η*: degradation efficiency of Orange II; *C*: concentration of Orange II before reaction; the unit is g·L^−1^; and *C*_0_: concentration of Orange II after the reaction; the unit is g·L^−1^.

A pseudo-primary kinetic model was used to fit the activated persulfate degradation reaction rate of Orange II, which was calculated as shown in Equation (2).

(2) $$In(\frac{C_{0}}{C})=kt$$

where *k*: rate constants for catalyzed reactions in min^−1^; and *t*: time at which the catalytic reaction proceeds in min.

### 2.4. DFT Calculation

The Quantum ESPRESSO (QE) code completed the first-principles calculation. The Perdew–Burke–Ernzerhof (PBE) exchange-correlation function was used to optimize the structure. In the framework of the plane wave, the basis was set to 500 eV. The convergence criteria of the self-consistent field (SCF) were set to 0.02 eV·Å^−1^ and 1.0 × 10^−6^ eV·atom^−1^ [21,22].

## 3. Results and Discussion

### 3.1. Structure and Morphology of Co_78_Si_8_B_14_/g-C_3_N_4_

**Figure 2:**
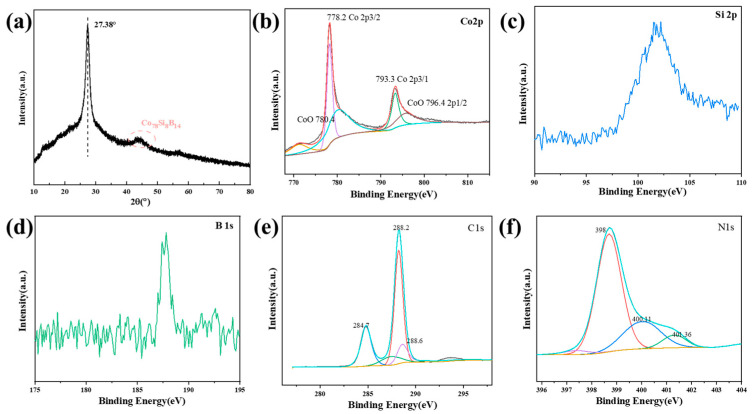
(**a**) XRD and (**b**) XPS patterns of Co_78_Si_8_B_14_/g-C_3_N_4_. (**c**) Spectrum of Si 2p, (**d**) Spectrum of B 1s, (**e**) Spectrum of C 1s, (**f**) Spectrum of N 1s.

Graphitic carbon nitride has a layered structure similar to graphite, and diffraction peaks corresponding to interlayer stacking and in-plane periodicity will appear in the XRD pattern. Two main characteristic peaks will appear near 2*θ* = 13° and 27°, corresponding to the (100) and (002) crystal planes of graphitic carbon nitride, respectively. Among them, the (100) plane reflects the in-plane periodicity of the triazine or heptazine ring structural units, while the (002) plane is related to the interlayer stacking of graphitic carbon nitride [23]. Amorphous materials usually show a broad scattering peak like a steamed bun in the XRD pattern. As shown in Figure 2, a distinct characteristic peak of g-C_3_N_4_ appears at 2*θ* = 27.38°, and an amorphous diffraction spectrum appears at around 2*θ* = 45°. The spectrum combines the characteristic peak changes of the two materials, initially confirming the successful preparation of the composite catalyst.

The XPS pattern of the sample is shown in Figure 2b–f, which proves the existence of Co, Si, B, C, and N. The corresponding binding energies are 778.2 eV, 102.3 eV, 188.1 eV, 284.4 eV, and 400.4 eV, respectively. Co 2p has an obvious spin–orbit splitting, two spectral peaks of Co 2p3/2 and Co 2p3/1 can be obtained, and the difference in binding energy between the two is 15.1 eV. The fitted peaks representing Co^2+^ appeared at 780.4 eV and 796.4 eV, and the corresponding oxide is CoO, indicating that there are unavoidable slight oxidation phenomena during the preparation and detection processes. In the Si 2p spectrum, at 102.3 eV, it is Si^2+^ [24]. For the B element, the binding energy at 188.1 eV is B_0_, indicating that the B-B bond is in the receiving band. The main peaks of N 1s of Co_78_Si_8_B_14_/g-C_3_N_4_ are located at 398 eV, 400.11 eV, and 401.36 eV. The binding energies at 398 eV and 400.11 eV belong to the nitrogen atoms in the sp_3_ C-N bond, and the nitrogen atoms combined with three carbon atoms in the amorphous C-N network. C 1s is composed of three binding energies, and the signals at 284.7, 288.2, and 288.6 eV correspond to the C-C bond, the sp^2^ hybridized C-N bond, and the sp^3^ hybridized C-N bond [25], respectively.

**Figure 3:**
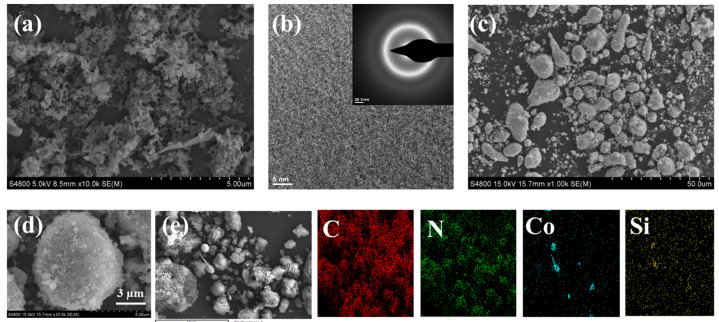
SEM and element distribution maps of (**a**) g-C_3_N_4_, (**b**) TEM pattern of Co_78_Si_8_B_14_, (**c**) Co_78_Si_8_B_14_/g-C_3_N_4_ (1000×), (**d**) Co_78_Si_8_B_14_/g-C_3_N_4_ (10,000×), and (**e**) element EDS.

Figure 3a is the SEM image of g-C_3_N_4_. It can be observed that g-C_3_N_4_ has a lamellar structure, and the thickness of the lamellae is relatively thin, within the nanometer range. The size of the lamellae is not completely uniform, and there is a certain distribution range. There is a certain distance between the lamellae, which may be caused by weak interaction between the lamellae or factors during the preparation process. Since amorphous materials do not have a long-range ordered crystal structure, the atomic arrangement is short-range ordered and long-range disordered [26]. Unlike the sharp diffraction spots of crystalline materials, the electron diffraction pattern of amorphous materials does not have obvious diffraction spots of crystal planes but appears in the form of a continuous halo with a relatively uniform intensity distribution. As shown in Figure 3b, when the Co_78_Si_8_B_14_ amorphous powder is subjected to electron diffraction under a transmission electron microscope, its diffraction pattern shows a diffuse halo. Figure 3 is the SEM image of Co_78_Si_8_B_14_/g-C_3_N_4_, showing particles of various shapes. The particles are stacked together to form a relatively dense structure. Further magnification is shown in Figure 3d, and a rough, spherical structure of about 5 um can be observed. The lamellar structure of the carbon nitride in the composite sample covers the amorphous particles.

Increasing the specific surface area can enhance the adsorption capacity of the catalyst for pollutants, providing more contact opportunities for the subsequent degradation reaction. A moderate specific surface area can reduce the risk of catalyst poisoning and maintain high catalytic activity [27]. The BET characterization of g-C_3_N_4_, Co_78_Si_8_B_14_, and Co_78_Si_8_B_14_/g-C_3_N_4_ powder materials shows that their specific surface areas are 65.49, 0.25, and 72.47 m^2^·g^−1^, respectively. As shown in Table 1.

### 3.2. Influence of Operative Parameters on Dye Degradation Analysis

#### 3.2.1. Influence of Systems on Dye Degradation Analysis

**Figure 4:**
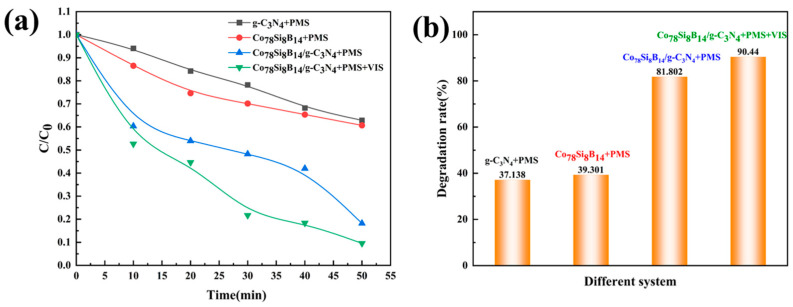
Degradation performance of Co_78_Si_8_B_14_/g-C_3_N_4_ composite catalyst in different systems: (**a**) normalized concentration change and (**b**) degradation rate change.

By comparing the degradation experiments of Orange II dye solution under the four conditions of Co_78_Si_8_B_14_ + PMS, g-C_3_N_4_ + PMS, Co_78_Si_8_B_14_/g-C_3_N_4_ + PMS, and Co_78_Si_8_B_14_/g-C_3_N_4_ + PMS + light, the catalytic ability of powder samples of different components and systems was evaluated. The experiment was carried out under the conditions of a dye concentration of 150 mg·L^−1^, no pH adjustment, a catalyst dosage of 0.2 g·L^−1^, a PMS concentration of 4 mM, and a reaction temperature of room temperature. The results are shown in Figure 4. Under the Co_78_Si_8_B_14_ + PMS and g-C_3_N_4_ + PMS systems, the degradation rates reached 39.30% and 37.12%, respectively, Co_78_Si_8_B_14_/g-C_3_N_4_ + PMS reached 81.80%, and Co_78_Si_8_B_14_/g-C_3_N_4_ + PMS + light reached the highest rate of 90.44%. The *k* values were fitted, as shown in Table 2. The correlation coefficient *R*^2^ is greater than 0.9, and In(C_t_/C_0_) has a good linear relationship with time t, indicating that the model is applicable. The reaction rate constants for the degradation of Orange II solution by g-C_3_N_4_ + PMS, Co_78_Si_8_B_14_ + PMS, Co_78_Si_8_B_14_/g-C_3_N_4_ + PMS, and Co_78_Si_8_B_14_/g-C_3_N_4_ + PMS + light were 0.01 min^−1^, 0.008 min^−1^, 0.026 min^−1^, and 0.043 min^−1^, respectively. The reaction rate constants of the degradation of Orange II solution by Co_78_Si_8_B_14_/g-C_3_N_4_ + PMS + VIS were 4.3 and 5.37 times those of the single g-C_3_N_4_ + PMS and Co_78_Si_8_B_14_ + PMS. Obviously, the degradation ability of the catalyst was improved after compounding to activate PMS with the assistance of visible light, which can be attributed to the larger specific surface area of the composite catalyst, which can provide more active sites and is beneficial for adsorbing dye molecules and activating PMS, thereby improving the degradation efficiency. At the same time, as a photocatalyst, g-C_3_N_4_ can use visible light to excite the photogenerated electron–hole pairs produced by the catalyst. These high-energy electrons can react with the PMS to generate more reactive oxygen species, such as hydroxyl radicals (·OH) and sulfate radicals (·SO_4_^−^) [28]. The synergy between photocatalysis and PMS activation improves the degradation efficiency of dyes. The optimum conditions obtained in this study were compared with other studies on the degradation of dyes (Table 3).

#### 3.2.2. Influence of Different Ratios on the Degradation Performance of Co_78_Si_8_B_14_/g-C_3_N_4_ Composite Catalyst

**Figure 5:**
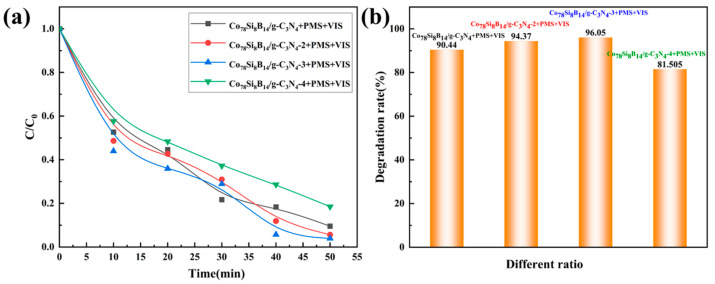
Degradation performance of Co_78_Si_8_B_14_/g-C_3_N_4_ composite catalyst under different ratios: (**a**) normalized concentration change and (**b**) degradation rate change.

In order to investigate the influence of the ratio of the two substances in the Co_78_Si_8_B_14_/g-C_3_N_4_ composite catalyst on the degradation effect, the degradation effects of Co_78_Si_8_B_14_/g-C_3_N_4_ at ratios of 1:1, 1:2, 1:3, and 1:4 were studied respectively. For the convenience of subsequent experimental descriptions, the composite catalysts were named Co_78_Si_8_B_14_/g-C_3_N_4_, Co_78_Si_8_B_14_/g-C_3_N_4_-2, Co_78_Si_8_B_14_/g-C_3_N_4_-3, and Co_78_Si_8_B_14_/g-C_3_N_4_-4 respectively. The experiment was carried out under the conditions of a dye concentration of 150 mg·L^−1^, no pH adjustment, catalyst dosage of 0.2 g·L^−1^, PMS concentration of 4 mM, and reaction temperature of room temperature. As shown in Table 4 and Figure 5, as the g-C_3_N_4_ content increases, the degradation rate shows a tendency to first increase and then decrease. When the ratio of Co_78_Si_8_B_14_/g-C_3_N_4_ is 1:3, the degradation effect is the best, reaching 96.05%, and the corresponding pseudo-first-order kinetic fitting *k* = 0.066 min^−1^. However, as the amount of compound is further increased, the degradation efficiency decreases to 81.5%, and the corresponding pseudo-first-order kinetic fitting *k* = 0.0279 min^−1^. It has been speculated that an excessively high composite amount may cause the active components to agglomerate on the surface of the carrier [33], resulting in the coverage or burial of some active sites and reducing the contact between the active sites and dye molecules, thereby decreasing the activity of the catalyst. Therefore, the optimal ratio of Co_78_Si_8_B_14_/g-C_3_N_4_-3 is selected for subsequent experiments.

#### 3.2.3. Influence of Light Intensity on the Degradation Performance of Co_78_Si_8_B_14_/g-C_3_N_4_ Composite Catalyst

**Figure 6:**
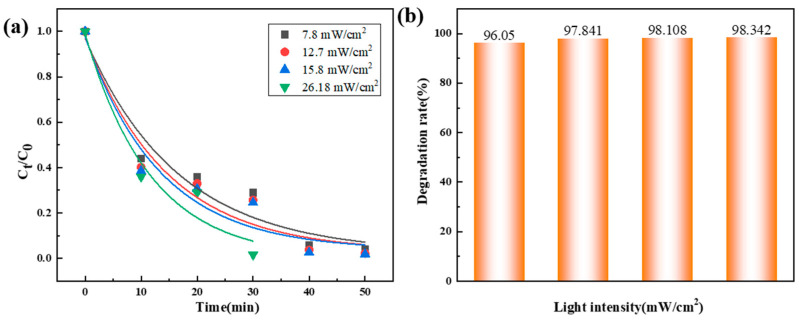
Degradation performance of Co_78_Si_8_B_14_/g-C_3_N_4_ composite catalyst under different light intensities: (**a**) normalized concentration change and (**b**) degradation rate change.

Figure 6 and Table 5 shows the effect of light intensity on the degradation effect of Orange II. As the light intensity increases from 7.8 mW·cm^−2^ to 26.18 mW·cm^−2^, the degradation rate improves significantly, and almost complete degradation is achieved in only 30 min at 26.18 mW·cm^−2^. It is speculated that light energy can improve the photochemical transformation between Co^0^, Co^2+^, and Co^3+^ [34], providing more sites for the reaction with PMS. At the same time, as the light intensity increases, the g-C_3_N_4_ semiconductor catalyst absorbs more photon energy, causing valence band electrons to transition to the conduction band and generating more photogenerated electron–hole pairs [35]. Strong light irradiation generates a large number of photogenerated carriers, and these carriers participate in the activation of PMS. In addition, the generated ·OH with a high redox potential (E_0_ = 2.7–2.8 V) can also enhance the degradation ability of the dye [34].

#### 3.2.4. Influence of the Dosage of Co_78_Si_8_B_14_/g-C_3_N_4_ Composite Catalyst on Degradation Performance

**Figure 7:**
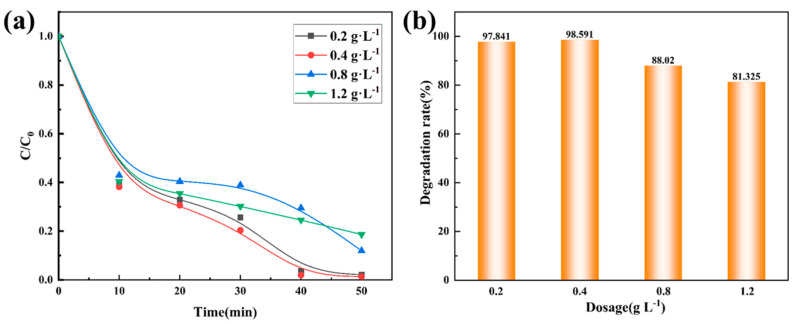
Effect of the amount of Co_78_Si_8_B_14_/g-C_3_N_4_ composite catalyst on the degradation performance. (**a**) change in normalized concentration, (**b**) change in degradation rate.

The catalyst can adsorb reactant molecules and allow them to react on the catalyst surface, thereby accelerating the degradation process. When the catalyst dosage is increased, more reactant molecules can react on the catalyst surface, thereby increasing the reaction rate. The experiment was carried out under the conditions of a dye concentration of 150 mg·L^−1^; no pH adjustment; catalyst dosages of 0.2, 0.4, 0.8, and 1.2 g·L^−1^; a PMS concentration of 4 mM; and a reaction temperature of room temperature. As shown in Figure 7 and Table 6, with the increase in the catalyst dosage, the degradation rate shows a trend of first increasing and then decreasing, which is consistent with the previously reported results. As mentioned earlier, the increase in dosage leads to an increase in active sites. However, when the catalyst concentration increases, it is speculated that the phenomenon of light scattering will intensify [36], resulting in a decrease in the number of incident photons and, thus, a decrease in the light-assisted reaction rate. High concentrations of the catalyst may generate more free radicals during the catalytic reaction process. If these free radicals cannot be consumed in time, they will react with the catalyst surface, resulting in changes in the catalyst structure and further reducing the stability of the catalyst [37].

#### 3.2.5. Influence of pH Value on the Degradation Performance of the Dosage of Co_78_Si_8_B_14_/g-C_3_N_4_ Composite Catalyst

**Figure 8:**
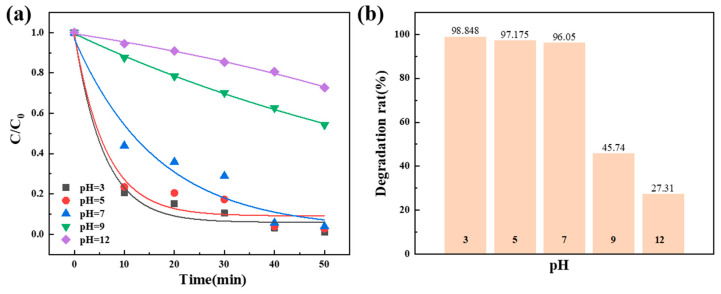
Effects of different pH conditions on the degradation performance of Co_78_Si_8_B_14_/g-C_3_N_4_ composite catalyst: (**a**) normalized concentration change and (**b**) degradation rate change.

To investigate the effect of pH value on the degradation performance of the dosage of Co_78_Si_8_B_14_/g-C_3_N_4_ composite catalyst, the experiment was carried out at room temperature. As shown in Figure 8, as pH changes from acidic to alkaline, the degradation performance of the Co_78_Si_8_B_14_/g-C_3_N_4_ catalyst for Orange II dye gradually decreases. The reaction is more likely to proceed under acidic conditions. When pH = 3, the degradation rate reaches 98%, but when pH = 12, the dye degradation rate is only 27.31%. This is related to the chemical stability or activity of the catalyst under pH conditions, and then the determination of the zero-charge point (PZC) is carried out.

**Figure 9:**
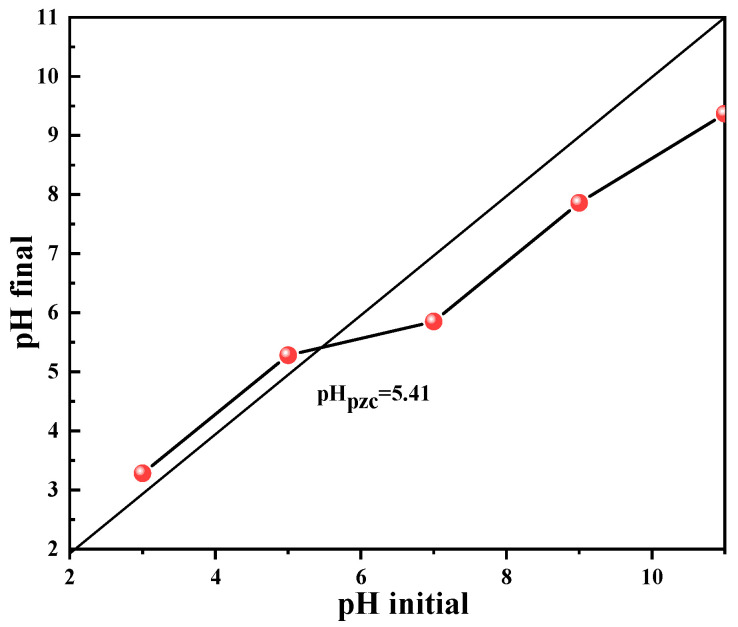
Zero-charge point of Co_78_Si_8_B_14_/g-C_3_N_4_ sample.

By dribbling an acid or base into a suspension containing solid particles, the pH of the solution is monitored. When approaching the PZC, the addition of a small amount of acid or base will cause a large change in pH value. Since the adsorption capacity of hydrogen ions and hydroxide ions on the solid surface is similar near the PZC, changing the concentration of acid and base in the solution will result in changes in the surface charge properties. Figure 9 shows the zero-charge point at pH_pzc_ = 5.41 of the Co_78_Si_8_B_14_/g-C_3_N_4_ sample. When the pH value of the solution is lower than the PZC, the catalyst surface is positively charged, and anions are more easily adsorbed to the surface. When the pH value is higher than the PZC, the solid surface is negatively charged, and cation adsorption predominates. As Orange II is an anionic dye, it is easier to promote the degradation reaction at pH = 5.41.

#### 3.2.6. Degradation Performance of Other Dyes

There are many types of pollutants in the actual environment. Evaluating the catalyst’s ability to degrade pollutants with different chemical structures and properties is the key to measuring its universality. Different types of organic dyes have different characteristics, such as chemical bond types, functional groups, molecular size, and polarity, and have different requirements for the active sites and reaction mechanisms of the catalyst [38].

**Figure 10:**
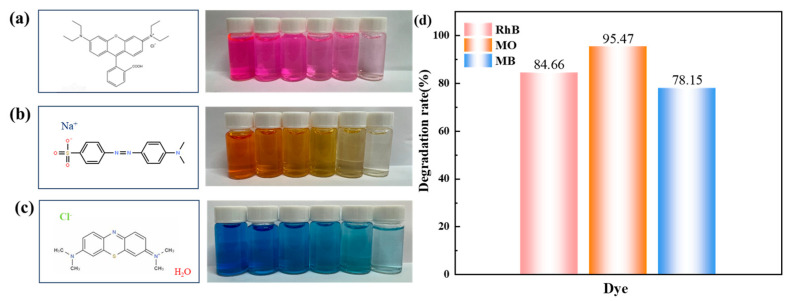
Color change of the three dye solutions: (**a**) RhB molecular formula and degradation color change, (**b**) AO7 molecular formula and degradation color change, (**c**) MB molecular formula and degradation color change, and (**d**) degradation rate change.

To test the universality of the catalytic material, triphenylmethane dye rhodamine B (RhB), azo dye methyl orange (MO), and methylene blue (MB) were selected as the target pollutants for degradation, and the calculation method of the degradation rate was the same as that for Orange II mentioned above. The experimental conditions were set as follows: the concentrations of RhB, MO, and MB solutions were all 20 mg·L^−1^, the pH value was not adjusted, the catalyst dosage was 0.8 g L^−1^, and the PMS concentration was 4 mM. As shown in Figure 10a, after 30 min of the reaction, the AO7 dye solution first changed significantly from bright orange to colorless and transparent. The degradation rate could reach 95.47% (Figure 10b). The decolorization rates of RhB and MB organic dyes could both reach more than 75%, confirming that the catalytic material has good universality.

### 3.3. Cycle Test

**Figure 11:**
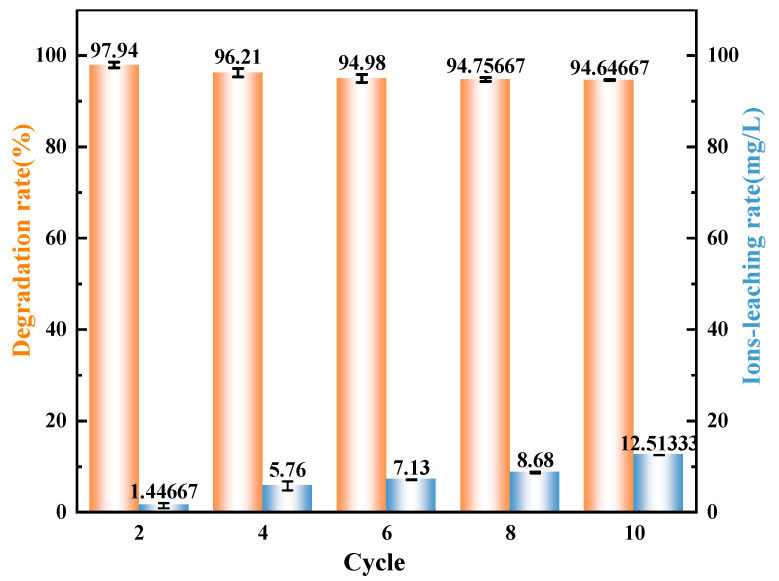
Cyclic test and ion leaching rate test.

During the reaction process, the performance of the catalytic material plays a crucial role. Among these, the loss situation of the active components is a key parameter for judging an excellent catalyst. Its presence and stability directly affect the catalytic efficiency and service life of the catalyst. Therefore, 10-cycle tests and Co^2+^ ion concentration were carried out on the composite catalyst. As can be seen from Figure 11, with the increase in the number of cycles, the change in the degradation rate is within 3%. After 10 cycles, the ion leaching concentration is 12.51 mg·L^−1^, and the ion loss rate is extremely low. This is attributed to the following reasons: Firstly, the sizes of Co_78_Si_8_B_14_ and/g-C_3_N_4_ are close to each other, which enables them to form a uniform and tightly combined composite structure, enhancing the interfacial interaction and effectively inhibiting the dissolution of metal ions such as Co through physical fixation and chemical bonding. Secondly, the introduction of B and Si elements further optimizes the electronic structure and surface chemical environment of the catalyst, enhances the coordination stability of Co, and forms an anti-corrosion protective layer on the catalyst surface, reducing the risk of metal ion loss. In addition, the layered structure of g-C_3_N_4_ not only provides physical coating protection for Co_78_Si_8_B_14_ particles but also further improves the stability of the catalyst by enhancing the electron transfer ability and antioxidant properties. During the catalytic process, Co can participate in the reaction through a reversible valence state cycle (Co^2+^/Co^3+^), thus avoiding the solubility problem caused by the accumulation of high-valent Co^3+^. Finally, the porous structure of the catalyst optimizes the distribution of PMS and prevents corrosive losses caused by excessive local concentration. Experimental characterizations show that these factors work together to enable the catalyst to maintain structural integrity and a low ion leaching rate after repeated use, showing excellent durability and long-term performance.

### 3.4. Degradation Mechanism Analysis

**Figure 12:**
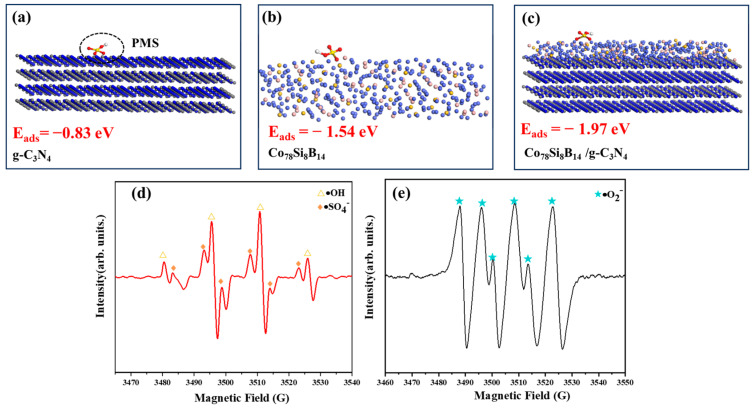
DFT adsorption energy calculations of (**a**) g-C_3_N_4_, (**b**) Co_78_Si_8_B_14_, and (**c**) Co_78_Si_8_B_14_/g-C_3_N_4_. (**d**,**e**) EPR detection curve.

The atomic structure, electronic structure, and other factors on the material surface can affect the interaction between the adsorbed molecules and the material surface [39]. DFT calculations were used to simulate the structures of each catalyst and compare the adsorption and activation abilities of different catalyst surfaces for PMS, as shown in Figure 12. The DFT calculation results show that the adsorption energies of PMS on the g-C_3_N_4_, Co_78_Si_8_B_14_, and Co_78_Si_8_B_14_/g-C_3_N_4_ catalysts are −0.83 eV, −1.54 eV, and −1.97 eV, respectively, indicating that PMS molecules are more likely to be adsorbed on the Co_78_Si_8_B_14_/g-C_3_N_4_ catalyst and form a stable configuration on the surface, which is conducive to the degradation reaction.

**Figure 13:**
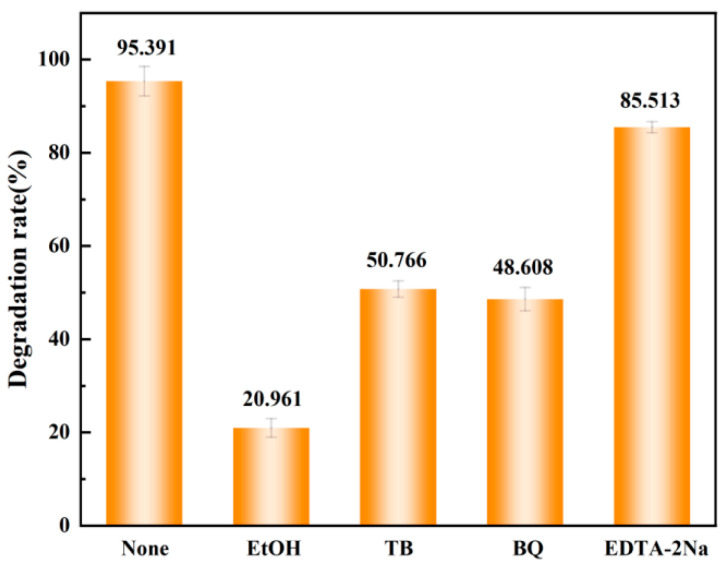
Trapping experiment.

As shown in Figure 13. After persulfate activation, various active species will be generated, such as sulfate radical (·SO_4_^−^), hydroxyl radical (·OH), singlet oxygen (^1^O_2_), superoxide radical (·O_2_^−^), etc. To determine the main active substances in this system, combined with EPR electron paramagnetic resonance detection and analysis, different free radical quenchers were selected for the experiment. Tert-butanol (TBA) was selected as the trapping agent for hydroxyl radicals (·OH), p-benzoquinone (BQ) was selected as the trapping agent for superoxide radicals (·O_2_^−^), and since visible light was used for assistance in the experiment, sodium ethylenediaminetetraacetate (EDTA-2Na) was also selected as the trapping agent for holes (h^+^), and ethanol (EtOH) was selected as the trapping agent for sulfate radicals (·SO_4_^−^) and ·OH. By adding the above different quenchers to the reaction system, the change in the degradation rate of Orange II was observed. When no trapping agent was added, the degradation rate of Orange II was 95.39%, and the addition of EtOH, TB, BQ, and EDTA-2Na reduced the degradation rate to 20.96%, 50.76%, 48.60%, and 85.51%, respectively. The experimental results show that in the system of the Co_78_Si_8_B_14_/g-C_3_N_4_ nanocomposite catalyst for the visible light-assisted activation of PMS to degrade Orange II, ·SO_4_^−^, ·O_2_^−^, and ·OH are the main active substances, and h^+^ plays an auxiliary role. In addition, the EPR characterization of Figure 12 also confirms the modified results [40].

During the process of the Co_78_Si_8_B_14_/g-C_3_N_4_ composite catalyst in the degradation of Orange II through the visible light-assisted activation of PMS, it demonstrates multiple synergy effects, thereby significantly improving catalytic efficiency, stability, and recyclability. Based on the above experimental results and existing research, the mechanism of the Co_78_Si_8_B_14_/g-C_3_N_4_ composite catalyst for the visible light-assisted activation of PMS to degrade Orange II is proposed. First, according to BET characterization and DFT calculations, it can be confirmed that the composite catalyst has a high specific surface area and adsorption energy. Secondly, the porous structure and nitrogen sites of g-C_3_N_4_ provide more adsorption sites for PMS molecules and Orange II molecules, enhancing the contact between reactants and the catalyst. Dye molecules and PMS are more likely to be adsorbed on the composite catalyst, thereby promoting the activation of PMS and the degradation of Orange II. At the same time, under light conditions, g-C_3_N_4_ has good light absorption performance, can effectively absorb visible light and stimulate the generation of electron–hole pairs, and electrons transition to the conduction band while leaving holes in the valence band. These excited electrons are transferred to the metal active center of Co_78_Si_8_B_14_ through the good conductivity of g-C_3_N_4_, promoting the decomposition of PMS. This significantly enhances the photocatalytic activity of the reaction. In addition, photogenerated holes can also directly oxidize Orange II molecules or react with water to generate hydroxyl radicals, further participating in the degradation process of Orange II.

**Figure 14:**
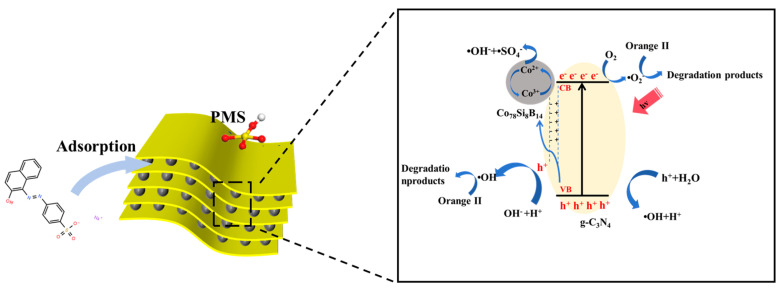
Co_78_Si_8_B_14_/g-C_3_N_4_ composite catalyst visible-light-assisted activation of PMS degradation of Orange II mechanism diagram.

In Co_78_Si_8_B_14_, amorphous cobalt atoms and atomic bonds can easily activate Co^2+^, and the inclusion of metal elements (Si, B) can improve amorphous cobalt-rich clusters and provide specific catalytic active sites, promoting the separation and transfer of electrons and holes. The metallic Co in Co_78_Si_8_B_14_ plays a key role in this process. Through electron transfer or coordination with PMS molecules, it activates PMS to generate strongly oxidizing free radicals, such as ·SO_4_^−^ and ·OH. These free radicals are the key active substances for the degradation of Orange II and have high oxidation ability. ·SO_4_^−^ free radicals degrade by extracting electrons and breaking covalent bonds in Orange II molecules, while ·OH further destroys the dye structure by oxidizing organic functional groups in Orange II, similar to the traditional Fenton reaction system. During the catalytic reaction process, Co_78_Si_8_B_14_ not only promotes the activation of PMS but also improves the stability and efficiency of the reaction through the reversible cycle transformation of Co^2+^/Co^3+^. The reversible valence state change of Co helps to avoid the accumulation of high-valent Co^3+^, thereby inhibiting metal dissolution and maintaining the long-term stability and catalytic activity of the catalyst. It is also mentioned in the literature that through the reversible valence state regulation of metals, the stability of the catalyst can be significantly improved. The stability and regeneration ability of the composite catalyst ensure its high efficiency and durability in multiple reaction cycles. The synergy between g-C_3_N_4_ and Co_78_Si_8_B_14_ enables the catalyst to exhibit higher performance in the degradation of Orange II through the visible light-assisted activation of PMS. The schematic diagram of the mechanism is shown in Figure 14. The layered structure of g-C_3_N_4_ provides physical protection for Co_78_Si_8_B_14_ particles, reduces the dissolution of metal ions, avoids the deactivation of the catalyst, and enhances its corrosion resistance, which is consistent with the research results of other metal-nitride composite materials in terms of catalytic stability.

In conclusion, the Co_78_Si_8_B_14_/g-C_3_N_4_ composite catalyst significantly improves the degradation efficiency of Orange II through the synergy of multiple mechanisms, such as light excitation, PMS activation, free radical generation, enhanced adsorption, and metal ion stabilization. The specific equations are as follows:

(3) $$g-C_{3}N_{4}+h\nu \to e^{-}+h^{+}$$

(4) $$h^{+}+H_{2}O\to \cdot OH+H^{+}$$

(5) $$h^{+}+\mathrm{PMS}\to \cdot {{\mathrm{SO}}_{4}}^{-}+{\mathrm{OH}}^{-}$$

(6) $${\mathrm{Co}}^{0}-{2e}^{-}\to {\mathrm{Co}}^{2+}$$

(7) $${\mathrm{Co}}^{0}{{+2\mathrm{HSO}}_{5}}^{-}\to {\mathrm{Co}}^{2+}+2{\mathrm{OH}}^{-}+2\cdot {{\mathrm{SO}}_{4}}^{-}$$

(8) $${\mathrm{Co}}^{3+}+{{\mathrm{HSO}}_{5}}^{-}\to {\mathrm{Co}}^{2+}+H^{+}+\cdot {{\mathrm{SO}}_{4}}^{-}$$

(9) $${\mathrm{Co}}^{3+}+{{\mathrm{HSO}}_{5}}^{-}\to {\mathrm{Co}}^{2+}+H^{+}+\cdot {{\mathrm{SO}}_{4}}^{-}$$

(10) $${\mathrm{Co}}^{2+}-e^{-}\to {\mathrm{Co}}^{3+}$$

(11) $$\cdot {{\mathrm{SO}}_{4}}^{-}/\cdot {O_{2}}^{-}/\cdot \mathrm{OH}/h^{+}+\mathrm{Orange}\;\mathrm{II}\;\mathrm{Dyes}\to \mathrm{Intermediate}\;\mathrm{product}\to {\mathrm{CO}}_{2}+H_{2}O$$

## 4. Conclusions

In this paper, the visible light-assisted activation of PMS for the degradation of Orange II over Co_78_Si_8_B_14_/g-C_3_N_4_ composite catalysts was investigated. The composite catalyst was prepared by the wet milling method, and its structure and morphology were analyzed using various characterization means, such as XRD and XPS. The results show that the catalyst integrates the properties of Co_78_Si_8_B_14_ amorphous alloy and g-C_3_N_4_ to form a composite structure with a large specific surface area, which is conducive to the enhancement of catalytic activity. The degradation performance test showed that the Co_78_Si_8_B_14_/g-C_3_N_4_ composite catalyst-activated PMS system had the highest degradation efficiency of 90.44% for Orange II under visible light assistance. The study of the effect of different factors revealed that the optimum ratio of Co_78_Si_8_B_14_/g-C_3_N_4_ was 1:3 when the degradation rate could reach 96.05%. Increased light intensity improves degradation efficiency. An optimum exists for the amount of catalyst. Acidic conditions are more favorable for the reaction. In addition, the composite catalyst also showed a good degradation effect on Rh B, methyl orange, and methylene blue dyes, with the degradation rate changing within 3% after 10 cycles, a low ion leaching concentration, and good stability. The degradation mechanism was explored by DFT calculations, EPR assay, and free radical trapping experiments, and it was found that PMS had the lowest adsorption energy on this catalyst, and the main active species of the system were ·SO_4_^−^, ·O_2_^−^, and ·OH. During the degradation process, the enhanced adsorption capacity, visible light photoexcitation, activation of PMS, free radical generation and metal ion stabilization, and other synergistic effects show a high efficiency of degradation performance, good stability, and wide applicability for the treatment of printing and dyeing wastewater treatment and provide a catalyst material with potential.

## Figures and Tables

**Table 1:** Analysis of specific surface area of samples.

Sample	Multipoint Specific Surface Area (m^2^·g^−1^)
g-C_3_N_4_	65.49
Co_78_Si_8_B_14_	0.25
Co_78_Si_8_B_14_/g-C_3_N_4_	72.47

**Table 2:** Pseudo-first-order kinetic fitting results of Co_78_Si_8_B_14_/g-C_3_N_4_ composite catalyst degradation in different systems.

Sample	*k* (min^−1^)	*R* ^2^
g-C_3_N_4_ + PMS	0.0101	0.99
Co_78_Si_8_B_14_ + PMS	0.0084	0.95
Co_78_Si_8_B_14_/g-C_3_N_4_ + PMS	0.0264	0.92
Co_78_Si_8_B_14_/g-C_3_N_4_ + PMS + VIS	0.0430	0.94

**Table 3:** Comparison of our study with other Fenton-like studies.

Catalyst	Dye	Time	Degradation	Ref.
Co_78_Si_8_B_14_/g-C_3_N_4_	Orange II	——	90.44%	This work
MIL-53(Al)@TiO_2_	Methylene Blue	240 min	95.00%	[29]
AsA	Methyl Orange	180 min	96.00%	[30]
Ag/ZnO	Methyl Orange	360 min	80.92%	[31]
Fe(III)	Reactive Black 5	60 min	80.00%	[32]

**Table 4:** *k* value of Co_78_Si_8_B_14_/g-C_3_N_4_ composite catalyst under different ratios.

Sample	*k* (min^−1^)	*R* ^2^
Co_78_Si_8_B_14_/g-C_3_N_4_ + PMS + VIS	0.0430	0.94
Co_78_Si_8_B_14_/g-C_3_N_4_-2 + PMS + VIS	0.0558	0.93
Co_78_Si_8_B_14_/g-C_3_N_4_-3 + PMS + VIS	0.0666	0.88
Co_78_Si_8_B_14_/g-C_3_N_4_-4 + PMS + VIS	0.0279	0.96

**Table 5:** Degradation fitting *k* value of Co_78_Si_8_B_14_/g-C_3_N_4_ composite catalyst under different light intensities.

Light Intensity (mW·cm^−2^)	*k* (min^−1^)	*R* ^2^
7.80	0.0666	0.91
12.70	0.0804	0.88
15.80	0.0850	0.87
26.18	0.1251	0.86

**Table 6:** Degradation fitting *k* value of Co_78_Si_8_B_14_/g-C_3_N_4_ composite catalyst dosage.

Dosage (g L^−1^)	*k* (min^−1^)	*R* ^2^
0.2	0.0804	0.85
0.4	0.0934	0.88
0.8	0.0286	0.92
1.2	0.0190	0.97

## Data Availability

The original contributions presented in this study are included in the article. Further inquiries can be directed to the corresponding author.
